# Genome-wide CRISPR Screen to Identify Genes that Suppress Transformation in the Presence of Endogenous *Kras*^G12D^

**DOI:** 10.1038/s41598-019-53572-w

**Published:** 2019-11-20

**Authors:** Jianguo Huang, Mark Chen, Eric S. Xu, Lixia Luo, Yan Ma, Wesley Huang, Warren Floyd, Tyler S. Klann, So Young Kim, Charles A. Gersbach, Diana M. Cardona, David G. Kirsch

**Affiliations:** 1 Department of Radiation Oncology, Duke University Medical Center, Durham, North Carolina 27708 USA; 2 Department of Pharmacology and Cancer Biology, Duke University Medical Center, Durham, North Carolina 27708 USA; 3 Medical Scientist Training Program, Duke University Medical Center, Durham, North Carolina 27708 USA; 4 Department of Biomedical Engineering, Duke University, Durham, North Carolina 27708 USA; 5 Duke Center for Genomic and Computational Biology, Duke University, Durham, North Carolina 27708 USA; 6 Department of Molecular Genetics and Microbiology, Duke University, Durham, North Carolina 27708 USA; 7 Department of Pathology, Duke University, Durham, North Carolina 27708 USA

**Keywords:** High-throughput screening, Sarcoma

## Abstract

Cooperating gene mutations are typically required to transform normal cells enabling growth in soft agar or in immunodeficient mice. For example, mutations in *Kras* and transformation-related protein 53 (*Trp53*) are known to transform a variety of mesenchymal and epithelial cells *in vitro* and *in vivo*. Identifying other genes that can cooperate with oncogenic *Kras* and substitute for *Trp53* mutation has the potential to lead to new insights into mechanisms of carcinogenesis. Here, we applied a genome-wide CRISPR/Cas9 knockout screen in *Kras*^G12D^ immortalized mouse embryonic fibroblasts (MEFs) to search for genes that when mutated cooperate with oncogenic *Kras* to induce transformation. We also tested if mutation of the identified candidate genes could cooperate with *Kras*^G12D^ to generate primary sarcomas in mice. In addition to identifying the well-known tumor suppressor cyclin dependent kinase inhibitor 2A (*Cdkn2a*), whose alternative reading frame product p19 activates *Trp53*, we also identified other putative tumor suppressors, such as F-box/WD repeat-containing protein 7 (*Fbxw7*) and solute carrier family 9 member 3 (*Slc9a3*). Remarkably, the TCGA database indicates that both *FBXW7* and *SLC9A3* are commonly co-mutated with *KRAS* in human cancers. However, we found that only mutation of *Trp53* or *Cdkn2a*, but not *Fbxw7* or *Slc9a3* can cooperate with *Kras*^G12D^ to generate primary sarcomas in mice. These results show that mutations in oncogenic *Kras* and either *Fbxw7* or *Slc9a3* are sufficient for transformation *in vitro*, but not for *in vivo* sarcomagenesis.

## Introduction

Cancers frequently arise when normal cells accumulate multiple gene mutations that results in transformation^1^. Oncogenic mutation of *KRAS* is identified in several cancer types^2–4^, including soft tissue sarcomas (STSs)^5^. KRAS-mutant tumors are heterogeneous in part because of co-mutation of other genes^6^. Therefore, understanding the pattern of genes co-mutated with *Kras* may provide novel insights into KRAS-mutant cancers with clinical implications^6,7^. Conditional activation of an endogenous *Kras*^G12D^ allele in mouse embryonic fibroblasts (MEFs) is sufficient to induce indefinite proliferation *in vitro* (immortalization), but further genetic alteration, such as mutation of *Trp53*, is required for full transformation to enable growth in soft agar or in nude mice^8^. *Trp53* serves as a transcriptional activator that regulates multiple genes^9^. However, the majority of *Trp53* canonical target genes are dispensable for its potent tumor suppression function^10^. Therefore, identification of critical downstream genes that can substitute for *Trp53* mutation and cooperate with oncogenic *Kras* to drive transformation can potentially lead to new insights into mechanisms of carcinogenesis, such as pathways by which *Trp53* suppresses cancer. The CRISPR/Cas9 system combines Cas9 nuclease activity with targeted single guide RNAs (sgRNAs) to achieve efficient genome editing at precise sites within DNA. Cas9 targeted to a coding gene creates double strand breaks that are repaired by non-homologous end-joining, which can introduce frameshift insertions and deletions (indels) that can result in loss-of-function (LOF) mutations^11^. Unbiased genome-wide CRISPR/Cas9 knockout screens have been applied to identify driver genes in different types of cancers, such as lymphoma^12^, liver tumors^13^, and breast cancer^14^, but to our knowledge have not been performed to seek genes that cooperate with oncogenic *Kras* for transformation.

Here, we performed an unbiased genome-wide CRISPR/Cas9 knockout screen in *Kras*^G12D^ immortalized MEFs to search for genes that can cooperate with oncogenic *Kras* to drive growth in soft agar and in nude mice. From this screen, we identified several candidate genes whose mutation results in transformation. In addition, we further tested whether mutating these genes together with *Kras*^G12D^ could drive sarcomagenesis *in vivo* using our established CRISPR/Cas9 *in vivo* editing method^15^. Although mutating some of these candidate genes led to transformation *in vitro* and tumor formation when cells were injected into nude mice, mutating these genes together with *Kras*^G12D^ failed to drive sarcomagenesis *in vivo*. This suggests that *in vitro* transformation screens may fail to fully capture all of the required elements for *in vivo* tumorigenesis^15^.

## Results

### Mutation *of Trp53* by CRISPR/Cas9 transforms *Kras*^G12D^ immortalized MEFs

**Figure 1:**
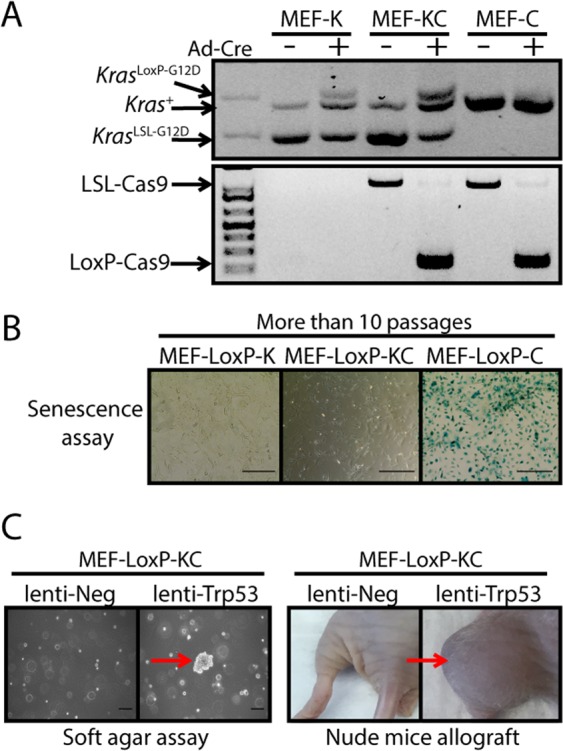
Immortalized MEFs expressing endogenous *Kras*^G12D^ are transformed by mutation of *Trp53*. (**A**) *Kras*^LSL-G12D^ MEFs (MEF-K), *Kras*^LSL-G12D^; *Rosa26*^LSL-Cas9-EGFP/+^ MEFs (MEF-KC), *Rosa26*^LSL-Cas9-EGFP/+^ MEFs (MEF-C) were infected with adenovirus expressing Cre (Ad-Cre) and then genotyped using PCR to confirm recombination of the floxed STOP cassette (LoxP). (**B**) Ad-Cre infected MEFs (MEF-LoxP-K, MEF-LoxP-KC, MEF-LoxP-C) were cultured for more than 10 passages and were stained with reagents for the β-Galactosidase senescence assay. (**C**) Immortalized MEF-LoxP-KC cells were transduced with lentivirus expressing either negative control sgRNA (lenti-Neg) or a sgRNA targeting *Trp53* (lenti-Trp53), and then seeded in soft agar or allografted in nude mice. MEF-LoxP-KC cells infected with *Trp53* sgRNA resulted in anchorage-independent growth in the soft agar assay and tumor formation in nude mice allografts (*n* = 2 or 3). The result is representative of at least two independent experiments. Scale bars = 100 µm.

To confirm MEFs can be immortalized by endogenous expression of *Kras*^G12D^, we isolated MEFs from *Kras*^LSL-G12D/+^ mice (MEF-K), *Rosa26*^LSL-Cas9-EGFP/+^ mice (MEF-C), as well as *Kras*^LSL-G12D/+^; *Rosa26*^LSL-Cas9-EGFP/+^ mice (MEF-KC). We then infected the MEFs with adenovirus to deliver Cre recombinase and activate either *Kras*^G12D^ in MEF-K (MEF-LoxP-K), Cas9 in MEF-C (MEF-LoxP-C) or both *Kras*^G12D^ and Cas9 in MEF-KC (MEF-LoxP-KC), as shown in Fig. 1A. The cells were cultured for greater than 10 passages and MEF-LoxP-K, MEF-LoxP-C, and MEF-LoxP-KC cells were stained for ∝-Galactosidase (SA-∝-Gal), a biomarker for cellular senescence. SA-∝-Gal activity was only significantly increased in the MEF-LoxP-C cells, indicating that only MEF-LoxP-C cells underwent senescence at late passage (Fig. 1B). In contrast, cells expressing oncogenic *Kras* (MEF-LoxP-K or MEF-LoxP-KC) were immortalized and did not stain for SA-∝-Gal. Furthermore, transduction of early passage MEF-LoxP-KC cells with a lentivirus delivering a sgRNA targeting *Trp53*, but not a negative control sgRNA, induced anchorage independent growth in soft agar and formed tumors when the transduced cells were allografted into nude mice (Fig. 1C). Thus, mutation of *Trp53* in MEF-LoxP-KC cells was sufficient for transformation. These results suggest that MEF-LoxP-KC cells represent a platform for a robust genome-wide CRISPR/Cas9 knockout screen to identify candidate genes whose mutations cooperate with oncogenic *Kras* to result in transformation.

### Genome-wide CRISPR/Cas9 knockout screen identifies *Trp53* as a dominant tumor suppressor

**Figure 2:**
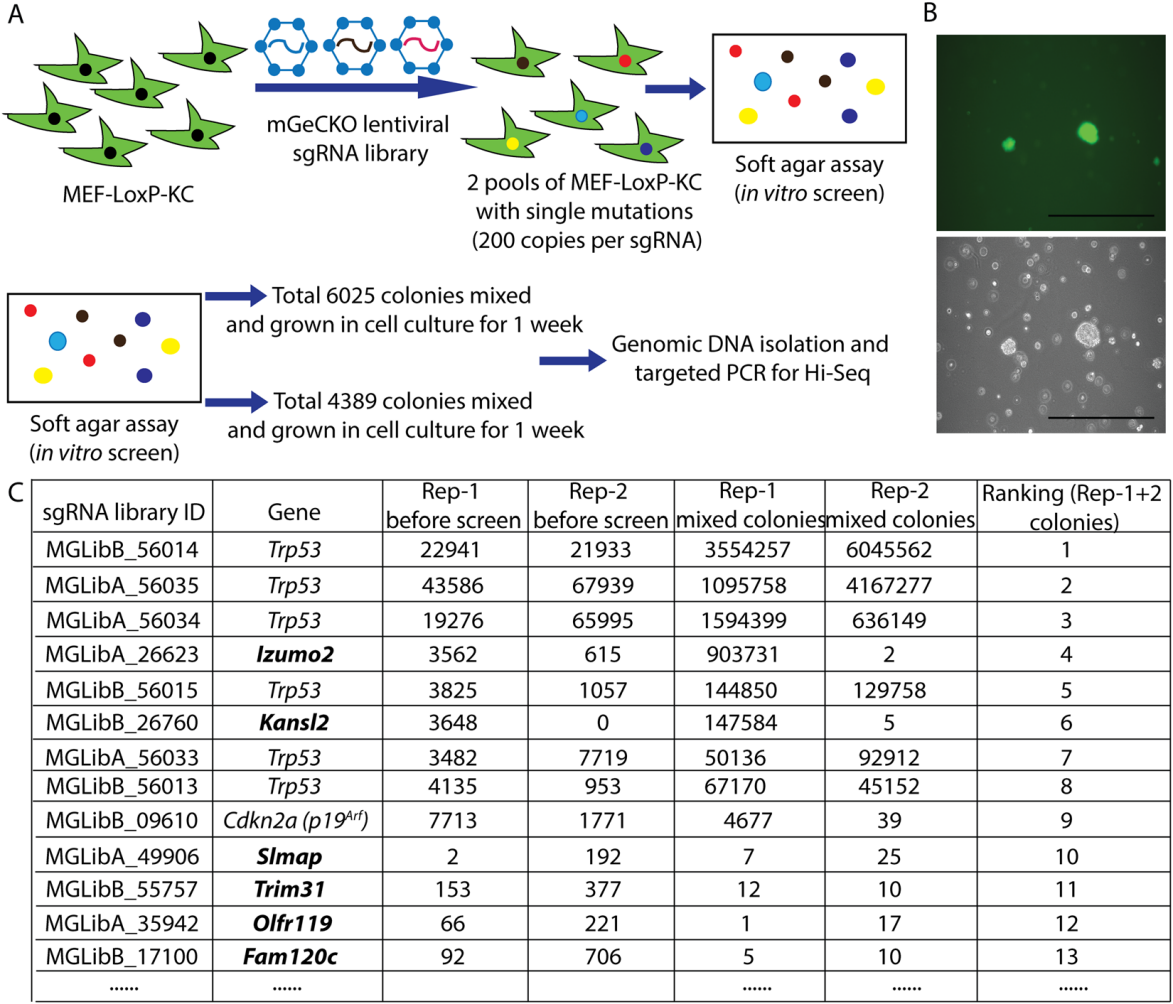
Screen with unbiased genome-wide CRISPR knockout library in MEF-LoxP-KC cells. (**A**) Schematic of the first unbiased genome-wide CRISPR knockout. (**B**) Representative colonies were identified in soft agar 3 weeks after transduced MEFs were seeded in soft agar. (**C**) The list of sgRNAs which were identified in colonies from the soft agar screen.

As shown in the schematic in Fig. 2A, low passage MEF-LoxP-KC cells were infected with a lentivirus expressing mouse genome-scale CRISPR/Cas9 knockout (GeCKO) sgRNA library at 0.2–0.4 multiplicity of infection (MOI). The library contains 103,209 sgRNAs targeting 20,611 mouse genes (six sgRNAs per coding gene and four sgRNAs per microRNA)^16,17^. Seven days after lentiviral transduction and puromycin selection, two independent mixed pools of stably transduced MEFs with approximately 200 copies per sgRNA were plated in soft agar to positively select transformed cells by anchorage-independent growth. Colonies were detected in soft agar after 3 weeks (Fig. 2B). 6025 colonies were counted in the first group and 4389 colonies were counted in the second group. Because of the large number of colonies that grew in the soft agar screen, for each screen we combined the colonies into a single pool and recovered cells following one additional week in culture. sgRNA cassettes were amplified from genomic DNA extracted from transduced MEFs before growth in soft agar and also from MEFs recovered after the soft agar screen. Then, amplicons of sgRNA cassettes were quantified by next-generation sequencing to identify sgRNAs. Both independent screens identified all 6 sgRNAs targeting *Trp53* (Fig. 2C). While *Trp53* sgRNAs were dominant in both soft agar screens, we also identified several other sgRNAs targeting *Izumo2*, *Kansl2*, *Cdkn2a*, *Slmap*, *Trim31*, *Olfr119*, and *Fam120c* in both screens. However, when MEF-LoxP-KC cells were transduced with lentiviruses expressing individual sgRNAs to *Izumo2*, or *Kansl2*, transduced cells did not consistently yield colonies in soft agar, which suggested that inactivation of each candidate gene alone is not sufficient to cooperate with oncogenic *Kras* to cause transformation (Fig. S3). Instead, sgRNAs targeting these candidate genes in the screen might be passenger sgRNAs identified in transformed MEFs which had also been targeted by a sgRNA to *Trp53*. Furthermore, this initial screen suggested that *Trp53* sgRNAs conferred a strong growth advantage during the one week expansion in culture prior to plating in soft agar, resulting in decreased representation of other sgRNAs and reduced sensitivity to identify less abundant and/or less potent sgRNAs from the soft agar colony screen.

### Genome-wide CRISPR/Cas9 sgRNA library without *Trp53* sgRNAs

**Figure 3:**
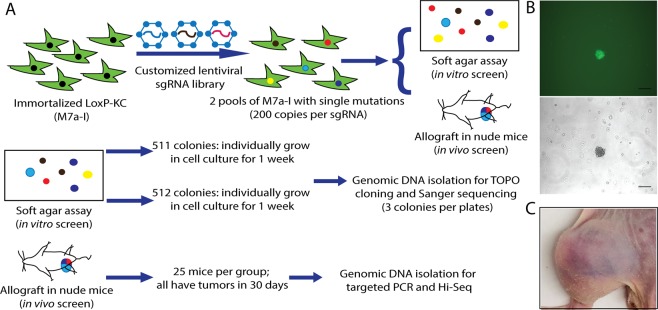
Screen with unbiased genome-wide CRISPR sgRNA knockout library without *Trp53* sgRNAs in MEF-LoxP-KC cells. (**A**) Schematic of screen with genome-wide CRISPR sgRNA library without *Trp53* sgRNAs. (**B**) Representative colonies were identified in soft agar 3 weeks after transduced MEFs were seeded in soft agar. (**C**) Representative tumor formation in nude mice allografts after transduced MEFs were intramuscularly injected and followed for 1 month.

Because our previous results demonstrated that *Trp53* sgRNAs dominated the soft agar screen, we generated a genome-wide CRISPR/Cas9 knockout sgRNA library without *Trp53* sgRNAs^18^. This sgRNA library is comprised of sequences from the mouse Brie library from Doench JG *et al*., not including *Trp53* sgRNAs, and also contains additional sgRNAs targeting microRNAs from the mGeCKO knockout sgRNA library^16,17^. Deep sequencing in Fig. S1 demonstrated good representation of sgRNAs in the library. Then, using this library we performed a second screen in low passage MEF-LoxP-KC cells. After stable transduction with lentivirus containing sgRNAs at 0.2–0.4 MOI, MEFs were plated in soft agar or allografted into nude mice to screen for transformed cells. We performed two independent screens. After transduced MEFs were cultured in soft agar for 3 weeks, the colonies were counted. There were 511 colonies in the first screen group, and 512 colonies in the second screen group (Fig. 3A). The individual colonies were each isolated and grown in cell culture for 1 week after which genomic DNA was isolated by TOPO cloning (Fig. 3B). Three clones from each colony were randomly picked for Sanger sequencing to identify integrated sgRNAs and an additional 7 clones were randomly picked for Sanger sequencing if more than one sgRNA was identified in the first 3 clones. Furthermore, each pool of MEFs transduced with the library was injected into the muscle of 25 nude mice with 2 million cells per mouse. After 30 days, all of the mice developed tumors at the injection site (Fig. 3C). Deep sequencing was performed to identify integrated sgRNAs from genomic DNA isolated from the tumors and compared to sgRNAs of the transduced MEFs before screening in soft agar or in nude mice.

### Screen with genome-wide CRISPR/Cas9 library without *Trp53* identifies multiple candidate genes that suppress transformation *in vitro*

**Figure 4:**
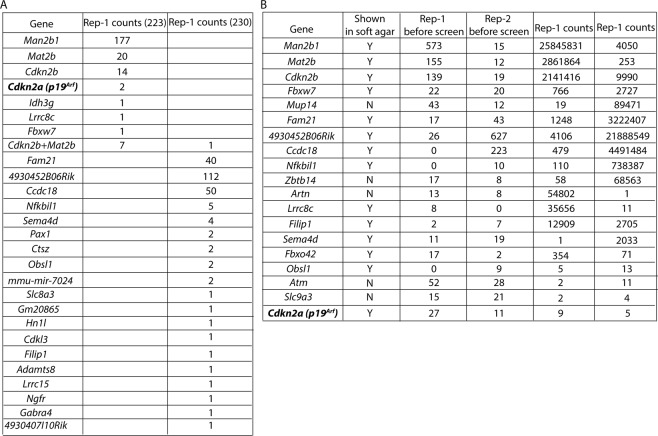
List of candidate genes that suppress transformation from screen with genome-wide CRISPR sgRNA library without *Trp53* sgRNAs. List of candidate genes identified by (**A**) the soft agar assay and from (**B**) nude mice allografts.

A total of 223 out of 511 colonies isolated from the first soft agar screen were able to grow in cell culture. Among these 223 colonies, Sanger sequencing from 216 of these cultured colonies identified only one sgRNA from each colony targeting 7 different genes, including *p19*^*Arf*^ encoded by *Cdkn2a* (Fig. 4A). Sanger sequencing of the remaining 7 cultured colonies identified two sgRNAs targeting *Cdkn2b* and *Mat2b*. In the second soft agar screen, a total 230 of 512 isolated colonies grew in cell culture. 229 of 230 cultured colonies contained only one sgRNA and Sanger sequencing results determined that a total of 19 individual sgRNAs targeting 19 different genes were identified (Fig. 4A). *p19*^*Arf*^ was not identified in this second screen (Fig. 4A). Sanger sequencing of the remaining colony identified two sgRNAs targeting *Cdkn2b* and *Mat2b* (Fig. 4A). Interestingly, none of the genes that were identified from colonies harboring a single targeting sgRNA were found in both soft agar screens, but colonies containing sgRNAs targeting both *Cdkn2b* and *Mat2b* were found in each of the soft agar screens.

Furthermore, deep sequencing was performed to identify the sgRNAs that were integrated into the genome of 50 tumors from the nude mice allograft screen (Fig. 4B). Thirteen sgRNAs were identified in the soft agar screen that were also found in the nude mice allograft screen. However, several sgRNAs were only identified in the nude mice allograft screen, including sgRNAs targeting *Mup14*, *Zbtb14*, *Artn*, *Atm*, *Fbxw7* and *Slc9a3*. Interestingly, *FBXW7* and *SLC9A3*, including several other candidates from the screen in nude mice, were identified as being prone to co-mutation with *KRAS* in the TCGA data set of all human cancers (Table S1). Overall, we identified several novel candidate genes whose mutation may result in transformation of *Kras*^G12D^ immortalized MEF-LoxP-KC.

### Mutation of *Slc9a3* transforms *Kras*^G12D^ immortalized MEF-LoxP-KC *in vitro*

**Figure 5:**
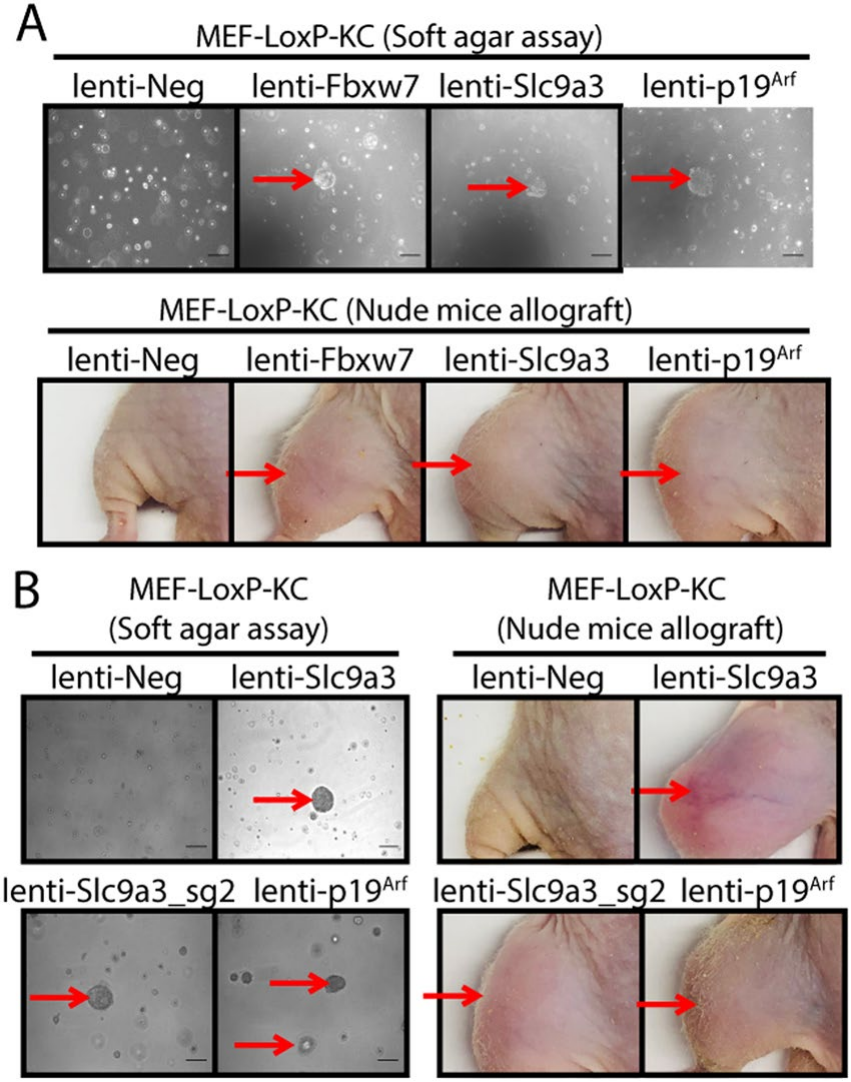
Individual validation of candidate genes that suppress transformation from the screen with genome-wide CRISPR sgRNA library without *Trp53* sgRNAs. (**A**) MEF-LoxP-KC cells transduced with a single candidate sgRNA were plated in soft agar or injected into the muscle of nude mice (*n* = 2 or 3). (**B**) MEF-LoxP-KC cells transduced with more than one sgRNA targeting *Slc9a3* were plated in soft agar assay or injected into the muscle of nude mice (*n* = 2 or 3). The result is representative of at least two independent experiments. Scale bars = 100 µm.

To further test the tumor suppressor function of the identified candidate genes, we transduced MEF-LoxP-KC cells with a lentivirus expressing each identified sgRNA targeting individual candidate genes identified from those screens (Table 1). Then, transduced cells were plated in soft agar and allografted into nude mice. Colony formation in soft agar and tumor growth in nude mice allografts were only detected in MEF-LoxP-KC cells transduced with lentivirus expressing sgRNA targeting either *p19*^*Arf*^, *Fbxw7* or *Slc9a3* (Fig. 5A). To further confirm the tumor suppression function of *Fbxw7* and *Slc9a3*, we transduced MEF-LoxP-KC cells with a different sgRNA targeting either *Fbxw7* or *Slc9a3*. While MEF-LoxP-KC cells transduced with a different sgRNA targeting *Slc9a3* consistently formed colonies in soft agar and tumors in nude mice allografts (Fig. 5B), we did not detect discernable colony formation in MEF-LoxP-KC cells transduced with a different sgRNA targeting *Fbxw7*. Thus, our results identify *Slc9a3* as a potential tumor suppressor whose mutation in *Kras*^G12D^-expressing MEFs induces transformation.

**Table 1:** The list of 21 candidate genes.

Target gene
*Kansl2*
*Izumo2*
*Slmap*
*Fbxw7*
*Filip1*
*Fbxo42*
*Man2b1*
*Cdkn2b*
*Mat2b*
*Fam21*
*4930452B06Rik*
*Obsl1*
*Sema4d*
*Ccdc18*
*Nfkbil1*
*Zbtb14*
*Mup14*
*Artn*
*Lrrc8c*
*Atm*
***Slc9a3***

### Expression of endogenous *Kras*^G12D^ and CRISPR/Cas9 *in vivo* editing of either *Trp53* or *p*19^*Arf*^, but not *Slc9a3*, induces primary sarcomas

**Figure 6:**
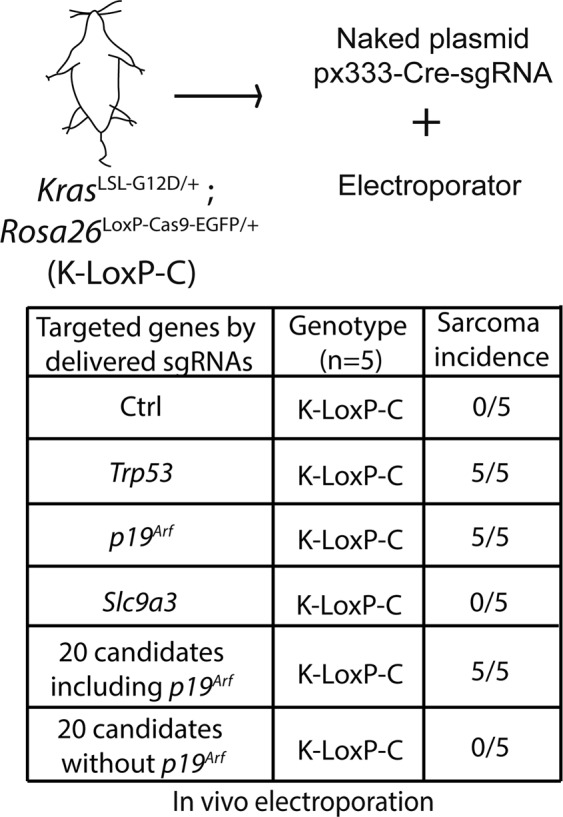
Testing candidate genes *in vivo*. Naked plasmid px333-Cre-sgRNAs containing different sgRNAs were electroporated *in vivo* into K-LoxP-C mice and the mice were followed up to 1 year for sarcoma formation (*n* = 5).

Because mutation of *Trp53* or *Cdkn2a* is sufficient to initiate sarcomagenesis with expression of endogenous *Kras*^G12D^ ^19^, we next investigated whether mutation of the identified candidate genes could induce primary sarcomas in *Kras*^LSL-G12D/+^; *Rosa26*^L^°^xP-Cas9-EGFP/+^ (K-LoxP-C) mice. We applied our recently reported *in vivo* electroporation method^15^ to intramuscularly deliver a naked plasmid px333-Cre-sgRNA that contains a sgRNA targeting only *Slc9a3* or a mixed pool of px333-Cre-sgRNAs that contain sgRNAs targeting the other 20 candidate genes that are from the screens (Table 1) either including *p19*^*Arf*^ *or* excluding *p19*^*Arf*^. In addition, K-LoxP-C mice that underwent *in vivo* electroporation with a non-targeting sgRNA served as a negative control and K-LoxP-C mice that underwent *in vivo* electroporated with a sgRNA targeting either *Trp53* alone or *p19*^*Arf*^ alone served as positive controls. All mice were followed for up to 1 year after injection. Interestingly, we only detected primary tumors in the positive control groups and the group of mice injected with the mixed pool of px333-Cre-sgRNAs including the *p19*^*Arf*^ sgRNA (Fig. 6). We examined the efficiency of CRISPR/Cas9 editing in primary tumors generated by the mixed pool of sgRNAs including the *p19*^*Arf*^ sgRNA, and we detected indels in multiple genes, including *p19*^*Arf*^ and *Fbxw7*. In addition, the primary tumors initiated by a sgRNA targeting *Trp53* or *p19*^*Arf*^ or the mixed pool of sgRNAs including the *p19*^*Arf*^ sgRNA have similar histologies on haematoxylin and eosin-stained sections (Fig. S4). Taken together, these data suggest that we achieved efficient CRISPR/Cas9 editing *in vivo* and that the mutation of *p19*^*Arf*^ was required to generate primary sarcomas (Fig. S2). Overall, our CRISPR/Cas9 *in vivo* editing results suggest that mutation of either *Trp53* or *p19*^*Arf*^ is necessary to cooperate with *Kras*^G12D^ to induce primary sarcomas.

## Discussion

We systematically searched for genes whose mutation together with expression of oncogenic *Kras* could transform MEFs. By using low passage *Kras*^G12D^ immortalized MEFs that constitutively express Cas9 from the *Rosa26* locus, we were able to efficiently transduce mouse genome-wide CRISPR/Cas9 knockout sgRNA libraries to perform a screen in cells with an identical genetic background. Because the *Trp53* sgRNAs dominated the first unbiased genome-wide CRISPR/Cas9 knockout screen, which limited the detection of other sgRNAs, we performed a second screen using a mouse genome-wide sgRNA library excluding *Trp53* sgRNAs. We identified several novel candidate genes using this library. Therefore, this genome-wide sgRNA library that excludes *Trp53* sgRNAs may be useful for other screens to search for tumor suppressor genes. For instance, this library could be potentially applied to low passage epithelial cells isolated from the K-LoxP-C mice to identify tumor suppressor genes in cell types other than MEFs.

In addition to the well-described tumor suppressor *Cdkn2a*, our screen identified several candidate genes such as *Fbxw7* and *Slc9a3*, which may cooperate with oncogenic Kras^G12D^ to transform cells. Tumor suppressor *FBXW7*, a component of the Skp1-Cullin1-Fbox E3 ubiquitin ligase complex, is frequently mutated in several types of human cancers^20^. Loss of *FBXW7* increases the expression of its oncogenic substrates, such as CCNE, MYC, NOTCH1, and JUN, which contribute to tumorigenesis^21^. Although we observed transformation of Cas9 expressing *Kras*^G12D^ immortalized MEFs with one sgRNA to *Fbxw7*, we did not observe transformation with a second sgRNA to *Fbxw7* (Fig. S3). Moreover, we failed to initiate sarcomas in K-LoxP-C mice (n = 5) electroporated with a sgRNA to *Fbxw7*. Therefore, it is conceivable that the single active *Fbxw7* sgRNA is driving transformation by mutating an off-target site in the genome. However, conditional deletion of *Fbxw7* accelerates *Kras*^G12D^ driven pancreatic cancer in a genetically engineered mouse model^21^. Moreover, human cancers in the TCGA database are prone to co-mutate *KRAS* and *FBXW7* (Table S1). Therefore, we cannot exclude the possibility that *Fbxw7* is a weak tumor suppressor, which would be sufficient to drive tumorigenesis with *Kras* mutation in a small number of mice if we utilized a much larger cohort of mice for the *in vivo* electroporation experiment. In addition, it is possible that co-mutation of *Kras* with certain *Fbxw7* mutations transforms cells and drives cancer development *in vivo* in a cell-type dependent manner.

*SLC9A3* is also known as sodium–hydrogen exchanger 3 (*NHE3*). The *SLC9* family of proteins play a critical role in electroneutral exchange of Na^+^ and H^+^ in mammalian cells and also regulate cell proliferation, apoptosis, and cell migration^22^. We found that two independent sgRNAs to *Slc9a3* transformed Cas9 expressing *Kras*^G12D^ immortalized MEFs. Moreover, *SLC9A3* is found co-mutated with *KRAS* in human cancers in the TCGA database (Table S1). However, electroporation of sgRNAs to *Slc9a3* into the muscle of *Kras* ^LSL-G12D/+^; *Rosa26*^LoxP-Cas9-EGFP/+^ (K-LoxP-C) mice (n = 5) failed to initiate sarcomagenesis. Similarly, with a sample size of 5 mice, we cannot exclude the possibility that *Slc9a3* is a weak tumor suppressor in the *in vivo* electroporation assay that may be sufficient to drive tumorigenesis. Taken together, these results suggest that mutation of *Kras* and *Slc9a3* may transform cells and drive tumorigenesis in a cell-type dependent manner. Therefore, in future studies, it would be interesting to test in mice if mutations in *Kras* and *Slc9a3* promote cancer development in tissues where these genes are frequently found co-mutated in human cancers (Table S1). Furthermore, *FBXW7* is significantly co-mutated with *KRAS* in colon cancer and *ATM* is significantly co-mutated with *KRAS* in pancreatic cancer (Table S4). Therefore, our list of candidate genes could be potentially validated in other types of cancer models.

Compared to sophisticated *in vivo* immunocompetent models, *in vitro* soft agar assays and *in vivo* cellular transplantation in immunocompromised mice have advantages for performing high throughput testing of a large number of candidate genes in a relatively short time. Therefore, *in vitro* soft agar assays and *in vivo* cellular transplantation in immunocompromised mice are valuable tools to identify candidate genes associated with tumorigenesis. However, our *in vivo* studies indicate that the results from the *in vitro* and transplant models may not be recapitulated in immunocompetent autochthonous models. Unfortunately, in many studies, including this one, mutating the candidate genes *in vivo* within a native tissue microenvironment fails to initiate cancer^23,24^. This discordance may simply reflect differences in the requirements between transformation of MEFs and *in vivo* sarcomagenesis. For example, it is possible that *in vivo* the immune system or other cells in the microenvironment play critical roles to prevent cancer initiation and progression^25^. One potential limitation of our *in vitro* CRISPR/Cas9 screen is that candidate genes that were mutated in immortalized MEFs may have created a growth disadvantage *in vitro* that would not occur *in vivo*. To address this limitation, in future experiments whole genome screening could be performed *in vivo* in the absence of *in vitro* culture. However, it is also possible that the candidate genes have the potential to promote tumorigenesis *in vivo* when mutations in these genes occur within a permissive cell type and developmental stage.

Our unbiased genome-wide approach using a CRISPR/Cas9 knockout screen for transformation in *Kras*^G12D^ immortalized MEFs successfully identified well-known tumor suppressors *Trp53* and *p19*^*Arf*^ and also candidate genes, such as *Fbxw7* and *Slc9a3*. We tested the potential tumor suppressor function of these genes *in vivo* using our recently reported CRISPR/Cas9 initiated *Kras*^G12D^-driven sarcoma model^15^. However, only mutation of *Trp53* or its upstream regulatory gene *p19*^*Arf*^ could cooperate with *Kras*^G12D^ to initiate primary sarcomas in skeletal muscle of adult mice. Therefore, the p19^Arf^-p53 pathway appears to be the critical tumor suppressor pathway to cooperate with oncogenic *Kras* to lead to *in vivo* sarcoma development. Because no *Trp53* target genes were identified in this screen, our results are consistent with a model where multiple *Trp53* downstream genes and pathways function simultaneously to suppress transformation^26^. To search for genes in addition to the p19^Arf^-p53 pathway that can promote sarcoma development, in the future we plan to perform an *in vivo* CRISPR/Cas9 screen in immune competent mice for genes that can cooperate with *Trp53* mutation. This approach has proven to be a useful tool to identify novel genes associated with tumorigenesis in mouse models of glioblastoma^27^, liver cancer^28^, and lung cancer^29^.

In conclusion, we identified *Fbxw7* and *Slc9a3* as genes that when mutated can transform *Kras*^G12D^ immortalized MEFs, but are unable to cooperate with oncogenic *Kras* to drive sarcomagenesis in the muscle of adult mice *in vivo*. Our mouse genome-wide sgRNA library excluding *Trp53* sgRNAs may be a useful tool for other researchers who perform screens where the dominant sgRNAs selected target *Trp53*.

## Methods

### Mice

*Kras*^LSL-G12D/+^ mice^30^ were a gift from T. Jacks (Massachusetts Institute of Technology). *Rosa26*^LSL-Cas9-EGFP/+^ mice^31^ were obtained from Jackson labs. *Rosa26*^LoxP-Cas9-EGFP/+^ mice were generated by methods described previously^15^. Athymic nude (nu/nu) mice (5 to 6 weeks old) for the allograft study were purchased from Taconic Biosciences and maintained in Duke University’s accredited animal facility. MEFs were transduced with lentivirus and 2 million cells were injected into the hind limbs of nude mice. All animal studies were performed in accordance with protocols approved by the Duke University Institutional Animal Care and Use Committee (IACUC).

### MEF isolation and transduction with adeno-cre

E13.5 to E14.5 embryos isolated from pregnant mice were used to isolate MEFs using a standard protocol. To activate expression of Cas9 from the *Rosa26* locus and to express Kras^G12D^ from the endogenous Kras locus, MEFs were infected with an adenovirus expressing Cre recombinase (University of Iowa).

### Molecular analysis of recombination

Recombination of the Cas9 allele and mutant *Kras* allele were assessed by PCR using methods described previously^15^ with primers in Supplementary Tables 2 and 3.

### Histology, immunohistochemistry, and tumor analysis

Tissues were harvested and fixed in 4% formalin and paraffin-embedded. Hematoxylin and eosin staining was performed using standard methods.

### Genomic DNA isolation, PCR amplification and indel analysis

Cells and tissues were harvested for genomic DNA isolation using DNeasy Blood & Tissue Kit (Qiagen, 69504). PCR amplification, re-annealing process and CRISPR/Cas9 induced indels on different targeted genes were performed using methods described previously^15^. PCR amplification of targeted genes was subjected to a re-annealing process to enable heteroduplex formation and indels were further detected using the Surveyor Mutation Detection Kit (IDT, 706021) following the standard protocol.

### CRISPR screens

Mouse Gecko v2 CRISPR libraries in the lentiguide-Puro vector were provided by Feng Zhang (MIT), made available through Addgene. A mouse genome-scale library without Trp53 sgRNAs was created with sgRNA sequences targeting coding genes and non-targeting controls taken from the Brie library and sgRNA sequences targeting miRNAs taken from the Gecko library for a total of 84,077 sgRNAs^16,18,32^. Oligonucleotide pools were ordered from Twist Bioscience and cloned into the lentiguide-Puro vector by Gibson assembly (NEB). Transformations of the assembled library were done into Endura competent cells (Lucigen) to provide 66-fold coverage of the library and sgRNA representation was verified by high-throughput sequencing. Lentivirus for the CRISPR libraries was prepared and MEFs were transduced under conditions to achieve MOI of 0.2–0.4 as described^33^. Cells were selected with puromycin for 48 hours and expanded for one week *in vitro* before plating into soft agar or injection into nude mice.

### Deep sequencing

Genomic DNA from cells or tumors was prepared by QiaAmp Blood Maxi Kit according to the manufacturer’s instructions.

The sgRNAs from each sample were PCR amplified using primers with Illumina adaptors and barcodes for multiplexing and sequenced on the Illumina HiSeq. 2500. Reads were trimmed and aligned to the library reference to generate read count files for each sgRNA using MAGeCK^34^.

### *In vivo* electroporation

All *in vivo* electroporations were performed using methods described previously^15^. 50 μl of 1 μg/ul naked DNA plasmids diluted in sterile saline was injected into the gastrocnemius using an insulin syringe. Then, a pair of needle electrodes was inserted into the muscle at the site of injection, and electric pulses were delivered using an electric pulse generator (Electro Square Porator ECM830; BTX, San Diego, CA).

### Senescence assay

Cells grown in tissue culture plates were subjected to a senescence assay using the manufacturer’s standard protocol (Cell Signaling Technology, 9860S).

### Soft agar assay

1.8% agar (BD Diagnostics, 204010) was made in de-ionized water and autoclaved. 2 x cell culture medium was made by dissolving 1 bag of High glucose DMEM powder (Thermo Fisher Scientific, 12800-017) and 1.85 g sodium bicarbonate (Thermo Fisher Scientific, 25080-094) in 500 ml of de-ionized water with pH adjusted to 6.8. The medium was sterilized by passage through a 0.2 μm filter. Sterilized medium was mixed with 20% FBS and 1% antibiotic-antimycotic (Thermo Fisher Scientific, 10091148). 2.5 ml of 0.6% agar medium consisting of 1.8% agar, 2x cell culture medium, and 1x cell culture medium as described previously^15^ was poured into 6-well plates. 0.25 ml medium containing 50,000 cells was mixed with 0.5 ml of 0.6% agar medium and was poured onto the top of the 0.6% agar medium in the plate. Plates were incubated at 37 ^o^C with 5% CO_2_ in a humidified cell-culture incubator and were fed with 0.5 ml of 1x cell culture medium every week. Colonies in the soft agar assay were counted, imaged, and picked for re-growth in tissue culture for 3 to 4 weeks.

### Co-mutation analysis

To analyze which mutations were enriched in *Kras* mutant cancers, we accessed the AACR GENIE dataset via cbioportal (http://genie.cbioportal.org/login.jsp). We then selected the “GENIE Cohort v6.1-public”. Then we used the “enhanced study view” feature and selected either “Colorectal Cancer”, “Non-Small Cell Lung Cancer”, “Sarcoma” or “Pancreatic Cancer”. We recorded the case ID’s associated with these cancer types, then returned to the home page (http://genie.cbioportal.org/) and chose “Query by Gene”, then input our the case ID’s of one of the cancers. Then, selecting for only Somatic Mutation, we queried for “KRAS”. On the following screen we then selected “enrichments” and chose the “mutation tab”, and selected the “Co-Occurrence” option on the screen. We then were able to sort by q-value and identify the top ten mutations with the highest tendency for co-occurrence for that cancer. We repeated these steps for each cancer type, and also for all cancer types combined.

## Supplementary information

Supplementary information
