# Supplementary material for: Genome-wide CRISPR Screen to Identify Genes that Suppress Transformation in the Presence of Endogenous KrasG12D
Source: Sci Rep. 2019 Nov 20;9:17220. doi: 10.1038/s41598-019-53572-w (PMC6868134; doi:10.1038/s41598-019-53572-w)
Supplement: Supplementary file 1 — Supplementary information [file 41598_2019_53572_MOESM1_ESM.pdf]

Title

Genome-wide CRISPR Screen to Identify Genes that Suppress Transformation in the Presence of Endogenous *Kras*^{G12D}

Authors

Jianguo Huang[a], Mark Chen[b,c], Eric S. Xu[a], Lixia Luo[a], Yan Ma[a], Wesley Huang[a], Warren Floyd[b,c], Tyler Klann[d,e], So Young Kim[f], Charles A. Gersbach[d,e], Diana M Cardona[g], David G. Kirsch[a,b]

[a] Department of Radiation Oncology, Duke University Medical Center, Durham, North Carolina, 27708

[b] Department of Pharmacology and Cancer Biology, Duke University Medical Center, Durham, North Carolina, 27708

[c] Medical Scientist Training Program, Duke University Medical Center, Durham, North Carolina, 27708

[d] Department of Biomedical Engineering, Duke University, Durham, North Carolina, 27708

[e] Duke Center for Genomic and Computational Biology, Duke University, Durham, North Carolina, 27708

[f] Department of Molecular Genetics and Microbiology, Duke University, Durham, North Carolina, 27708

[g] Department of Pathology, Duke University, Durham, North Carolina, 27708

Corresponding author

David G. Kirsch

Duke University Medical Center, DUMC Box 91006, Durham, North Carolina 27708

Tel: 919-68-8605, E-mail: david.kirsch@duke.edu

Supplementary Information

Supplementary Figure. S1: Deep sequencing of each sgRNA represented in the customized library.

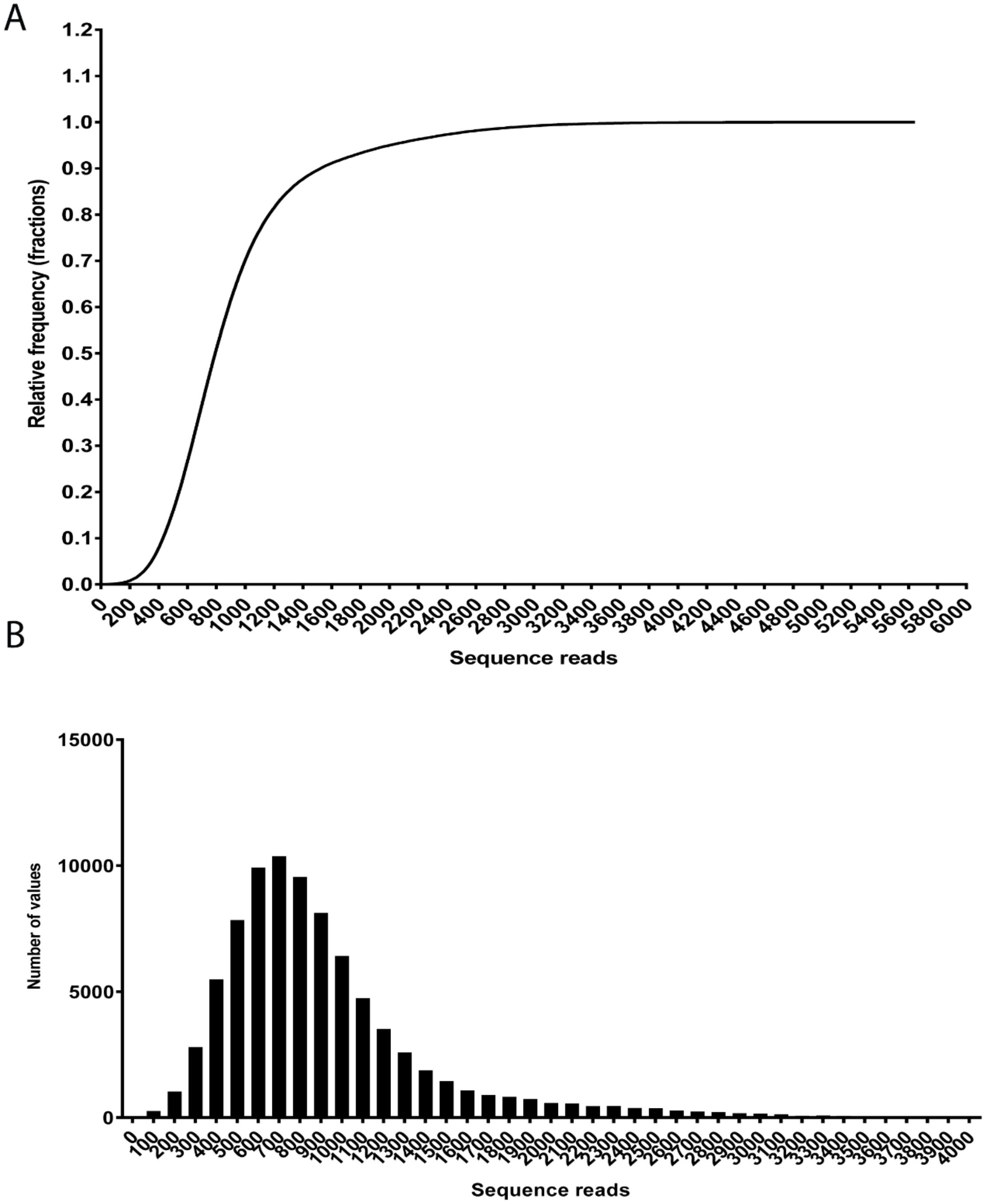

Supplementary Figure S2: (A) Surveyor assay on Tumor #1844. (B) Surveyor assay on Tumor #1846.

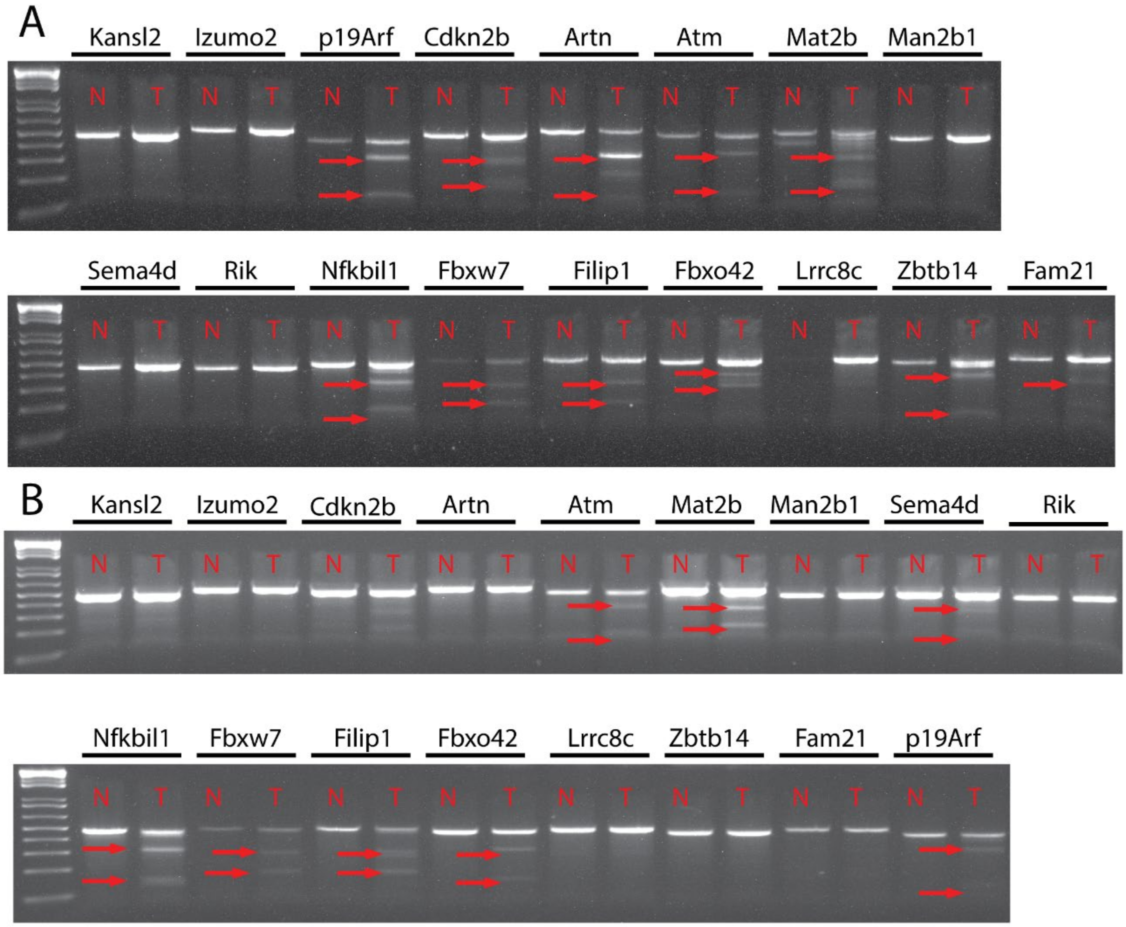

Supplementary Figure S3. (A) Surveyor assay on MEF-LoxP-KC infected with lentivirus that expresses a second sgRNA targeting *Fbxw7*, *Kansl2*, or *Izumo2*. The gel image of the marker and the gel image of the surveyor assay on lenti-Neg and lenti-Fbxw7 sg-2 were cropped from the same gel which is shown in **Supplementary Figure S5**. (B) MEF-LoxP-KC infected with lentivirus that expresses a second sgRNA targeting *Fbxw7*, *Kansl2*, or *Izumo2* failed to form colonies in soft agar.

A

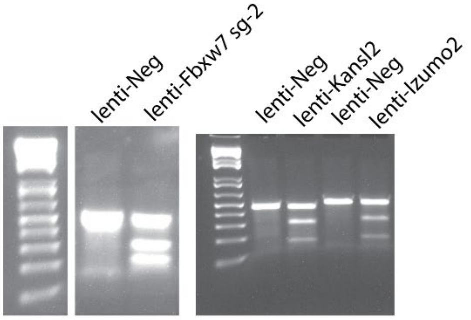

B

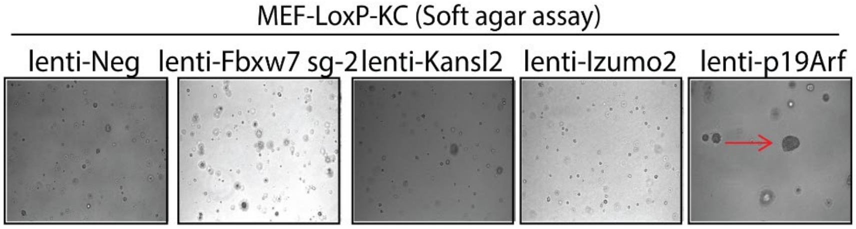

Supplementary Figure S4. Eight of the primary tumors generated in K-LoxP-C mice via IVE that delivered Cre recombinase and *Trp53* sgRNA, or *p19*^{Arf} sgRNA or mixed pool of sgRNAs targeting 20 candidates including *p19*^{Arf} were subject to histopathological analysis with haematoxylin and eosin staining. Scale bars, 200 μm.

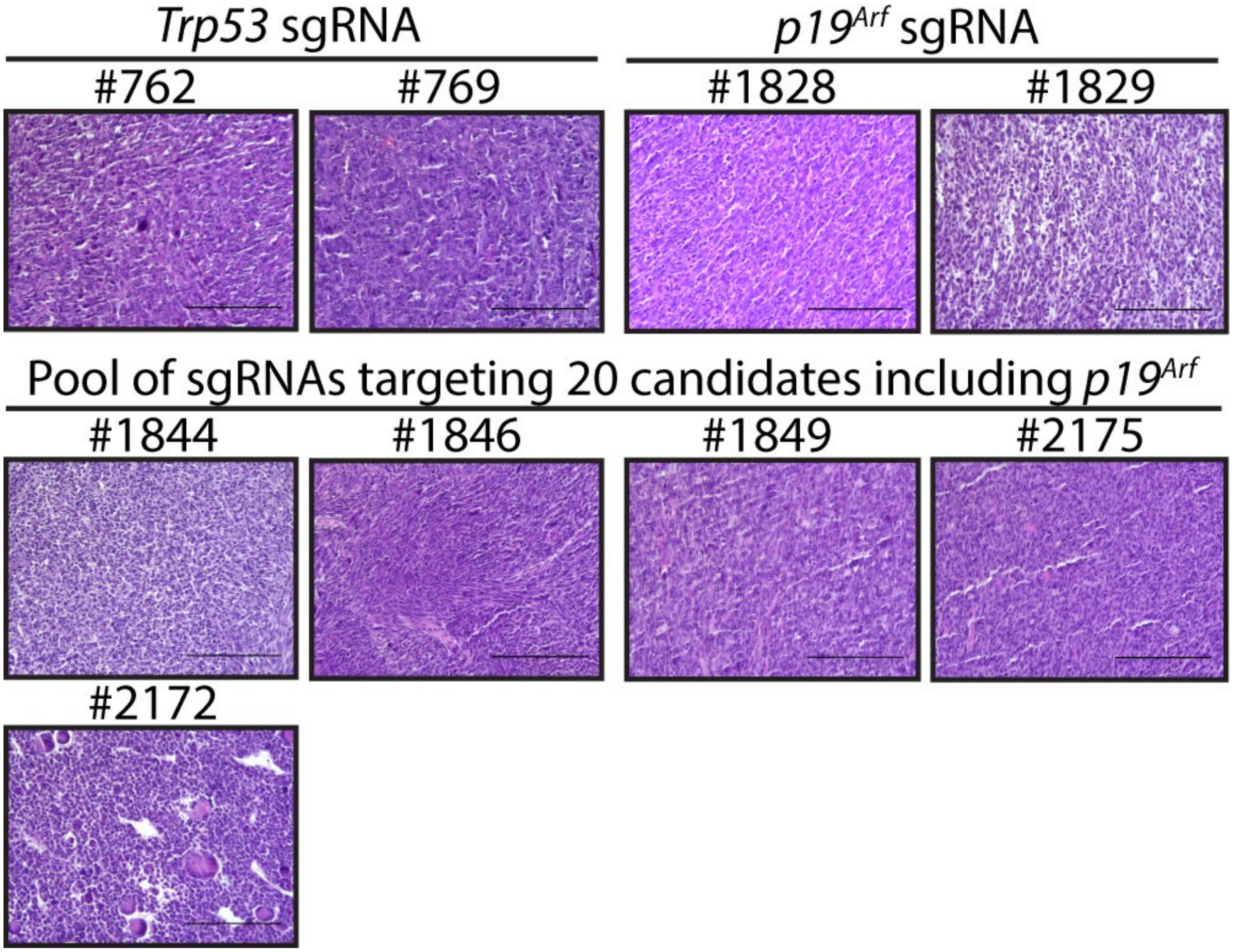

Supplementary Figure S5. All the uncropped images.

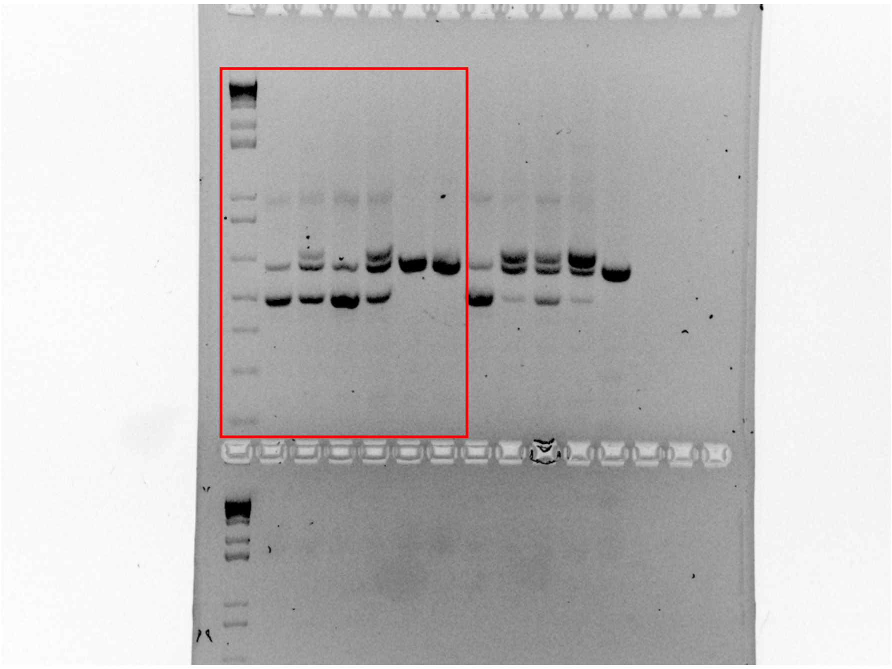

Figure 1A: Kras genotyping PCR.

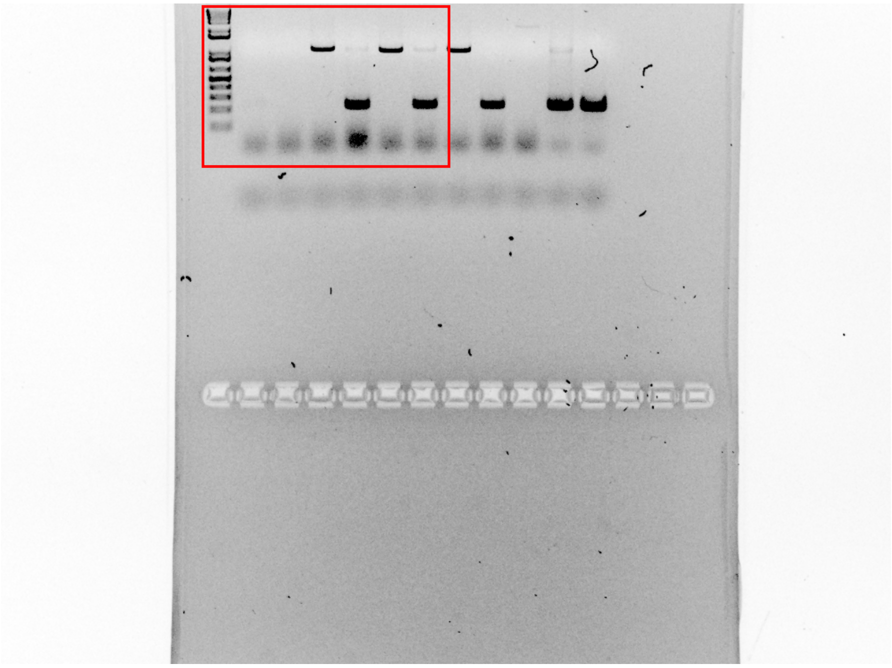

Figure 1A: Cas9 genotyping PCR.

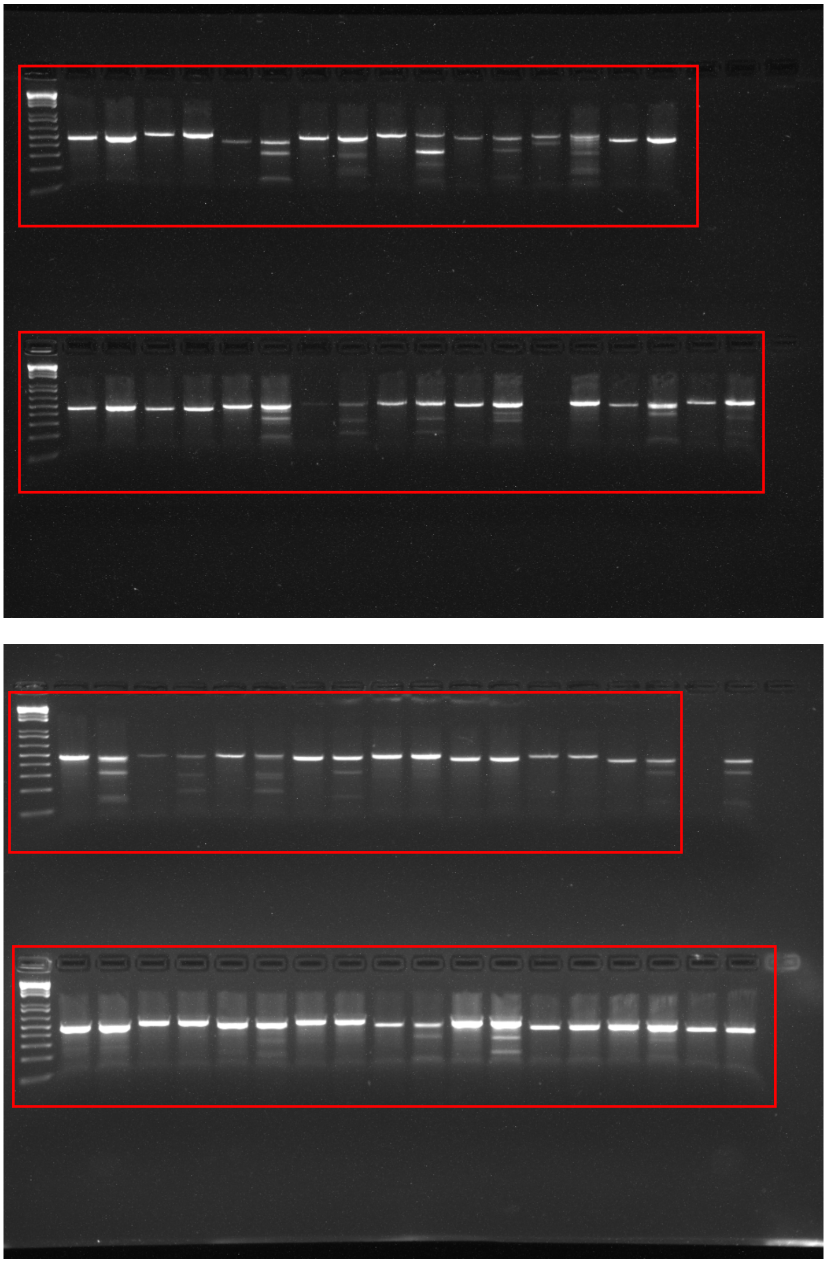

Figure S2: Surveyor assay gel images.

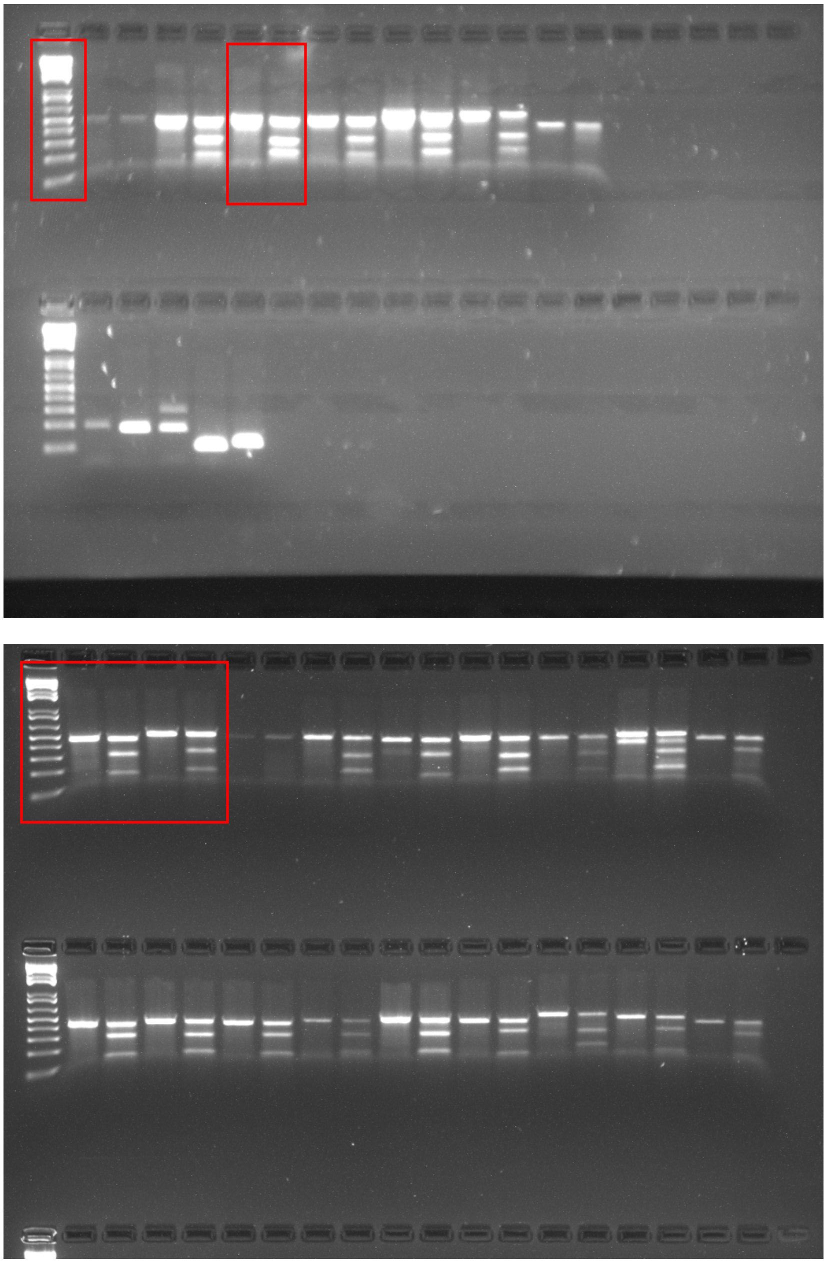

Figure S3: Surveyor assay gel images.

Supplementary Table S1
Correlation of *FBXW7* and *SLC9A3* with KRAS in all human cancer patients in the TCGA database.

Gene A	Gene B	Mutation Tendency: Co-occurrence/Mutual exclusivity (N/A: Not Applicable)	p-Value	Adjusted p-Value
KRAS	***FBXW7***	Co-occurrence	< 0.001	< 0.001
KRAS	***SLC9A3***	Co-occurrence	< 0.001	< 0.001
KRAS	*KANSL2*	Co-occurrence	< 0.001	< 0.001
KRAS	*IZUMO2*	Co-occurrence	0.31	0.31
KRAS	*SLMAP*	Co-occurrence	< 0.001	< 0.001
KRAS	*FILIP1*	Co-occurrence	< 0.001	< 0.001
KRAS	*FBXO42*	Co-occurrence	< 0.001	< 0.001
KRAS	*MAN2B1*	Co-occurrence	< 0.001	< 0.001
KRAS	*CDKN2B*	Co-occurrence	0.08	0.08
KRAS	*MAT2B*	Co-occurrence	< 0.001	< 0.001
KRAS	*FAM21*	N/A		
KRAS	*4930452B06RIK*	N/A		
KRAS	*OBSL1*	Co-occurrence	< 0.001	< 0.001
KRAS	*SEMA4D*	Co-occurrence	< 0.001	< 0.001
KRAS	*CCDC18*	Co-occurrence	< 0.001	< 0.001
KRAS	*NFKBIL1*	Co-occurrence	< 0.001	< 0.001
KRAS	*ZBTB14*	Co-occurrence	< 0.001	< 0.001
KRAS	*MUP14*	N/A		
KRAS	*ARTN*	Co-occurrence	< 0.001	< 0.001
KRAS	*LRRC8C*	Co-occurrence	< 0.001	< 0.001
KRAS	*ATM*	Co-occurrence	< 0.001	< 0.001

Supplementary Table S2

sgRNA sequences.

Target gene	gRNA ID	Target sequence (5' to 3')
Trp53	gTrp53	GTGTAATAGCTCCTGCATGG
Negative	gNegtive	GCGAGGTATTCGGCTCCGCG
p19Arf	gp19Arf	GGGCCGCCCACTCCAAGAGA
Kansl2	gKansl2	TTGATCACTGTCTATGCTAT
Izumo2	gIzumo2	GTCTGCAGTCGTGGATCAAC
Slmap	gSlmap	ATACTCACTCTGAACGAAGT
Fbxw7	gFbxw7 sg-1	GTGTTGCTGAACATGGTACA
Fbxw7	gFbxw7 sg-2	GTATGTCACAGATTCTAACG
Filip1	gFilip1	GTGGAGAGGGAAATCAACCG
Fbxo42	gFbxo42	GTCCTTGTACACGACCAGAG
Man2b1	gMan2b1	AGCTCCCAGAGGTAACACGT
Cdkn2b	gCdkn2b	GTCGTGCACAGGTCTGGTAA
Mat2b	gMat2b	GCTGCTTCCCAGCTGAATGT
Fam21	gFam21	ATTATGCAGACGACAATGTG
4930452B06Rik	gRik	CTGGCAGACGGAATAACCTA
Obsl1	gObsl1	TGTATGTGAGACCCGAGATG
Sema4d	gSema4d	GGCTGGATTCAGGACATGAG
Ccdc18	gCcdc18	AAAGGAGCACAGTTTACAAG
Nfkbil1	gNfkbil1	AGTGGCAGGAGGTCATCGGG
Zbtb14	gZbtb14	TTGCCGTCCTCTTGACTCGG
Mup14	gMup14	CTTCTGCATGGACACACACT
Artn	gArtn	AGGGCTAGAACAGCTAGGGT
Lrrc8c	gLrrc8c	TGGTTGACAAATCTGCTGCG
Atm	gAtm	TAAGTCATATAGGAAGCCGA
Slc9a3	gSlc9a3 sg-1	CTTCATAGTGTAGCGCACAG
Slc9a3	gSlc9a3 sg-2	CATAGTGTAGCGCACAGTGG

Supplementary Table S3
Primer sequences.

Primer ID	Sequence (5' to 3')	Use
gTrp53 5.1	CACCGTGTAATAGCTCCTGCATGG	Cloning gTrp53
gTrp53 3.1	AAACCCATGCAGGAGCTATTACAC	
gNegtive 5.1	CACCGGCGAGGTATTCGGCTCCGCG	Cloning gNegative
gNegtive 3.1	AAACCGCGGAGCCGAATACCTCGCC	
gp19Arf 5.1	CACCGGGGCCGCCCACTCCAAGAGA	Cloning gp19Arf
gp19Arf 3.1	AAACTCTCTTGGAGTGGGCGGCCCC	
gKansl2 5.1	CACCGTTGATCACTGTCTATGCTAT	Cloning gKansl2
gKansl2 3.1	AAACATAGCATAGACAGTGATCAAC	
gIzumo2 5.1	CACCGgtctgcagTCGTGGATCAAC	Cloning gIzumo2
gIzumo2 3.1	AAACGTTGATCCACGActgcagacC	
gSlmap 5.1	CACCGATACTCACTCTGAACGAAGT	Cloning gSlmap
gSlmap 3.1	AAACACTTCGTTCAGAGTGAGTATC	
gFbxw7 sg-1 5.1	CACCGGTGTTGCTGAACATGGTACA	Cloning gFbxw7 sg-1
gFbxw7 sg-1 3.1	AAACTGTACCATGTTCAGCAACACC	
gFbxw7 sg-1 5.1	CACCGGTATGTCACAGATTCTAACG	Cloning gFbxw7 sg-2
gFbxw7 sg-2 3.1	AAACCGTTAGAATCTGTGACATACC	
gFilip1 5.1	CACCGGTGGAGAGGGAAATCAACCG	Cloning gFilip1
gFilip1 3.1	AAACCGGTTGATTTCCCTCTCCACC	
gFbxo42 5.1	CACCGGTCCTTGTACACGACCAGAG	Cloning gFbxo42
gFbxo42 3.1	AAACCTCTGGTCGTGTACAAGGACC	
Man2b1 5.1	CACCGAGCTCCCAGAGGTAACACGT	Cloning gMan2b1
Man2b1 3.1	AAACACGTGTTACCTCTGGGAGCTC	
gCdkn2b 5.1	CACCGGTCGTGCACAGGTCTGGTAA	Cloning gCdkn2b
gCdkn2b 3.1	AAACTTACCAGACCTGTGCACGACC	
gMat2b 5.1	CACCGGCTGCTTCCCAGCTGAATGT	Cloning gMat2b
gMat2b 3.1	AAACACATTCAGCTGGGAAGCAGCC	
gFam21 5.1	CACCGATTATGCAGACGACAATGTG	Cloning gFam21
gFam21 3.1	AAACCACATTGTCGTCTGCATAATC	
gRik 5.1	CACCGCTGGCAGACGGAATAACCTA	Cloning gRik
gRik 3.1	AAACTAGGTTATTCCGTCTGCCAGC	
gObsl1 5.1	CACCGTGTATGTGAGACCCGAGATG	Cloning gObsl1
gObsl1 3.1	AAACCATCTCGGGTCTCACATACAC	
gSema4d 5.1	CACCGGGCTGGATTCAGGACATGAG	Cloning gSema4d
gSema4d 3.1	AAACCTCATGTCCTGAATCCAGCCC	
gCcdc18 5.1	CACCGAAAGGAGCACAGTTTACAAG	Cloning gCcdc18
gCcdc18 3.1	AAACCTTGTAAACTGTGCTCCTTTC	
gNfkbil1 5.1	CACCGAGTGGCAGGAGGTCATCGGG	Cloning gNfkbil1

gNfkbil1 3.1	AAACCCCGATGACCTCCTGCCACTC	
gZbtb14 5.1	CACCGTTGCCGTCCTCTTGACTCGG	Cloning gZbtb14
gZbtb14 3.1	AAACCCGAGTCAAGAGGACGGCAAC	
gMup14 5.1	CACCGCTTCTGCATGGACACACACT	Cloning gMup14
gMup14 3.1	AAACAGTGTGTGTCCATGCAGAAGC	
gArtn 5.1	CACCGAGGGCTAGAACAGCTAGGGT	Cloning gArtn
gArtn 3.1	AAACACCCTAGCTGTTCTAGCCCTC	
gLrrc8c 5.1	CACCGTGGTTGACAAATCTGCTGCG	Cloning gLrrc8c
gLrrc8c 3.1	AAACCGCAGCAGATTTGTCAACCAC	
gAtm 5.1	CACCGTAAGTCATATAGGAAGCCGA	Cloning gAtm
gAtm 3.1	AAACTCGGCTTCCTATATGACTTAC	
gSlc9a3 sg-1 5.1	CACCGCTTCATAGTGTAGCGCACAG	Cloning gSlc9a3 sg-1
gSlc9a3 sg-1 3.1	AAACCTGTGCGCTACACTATGAAGC	
gSlc9a3 sg-2 5.1	CACCGCATAGTGTAGCGCACAGTGG	Cloning gSlc9a3 sg-2
gSlc9a3 sg-2 3.1	AAACCCACTGTGCGCTACACTATGC	
gTrp53 svg 5.1	TGCCGAACAGGTGGAATATC	Amplify Trp53 locus for Surveyor
gTrp53 svg 3.1	CATCCTGACTGTGTGTAAACTAGGCCCC	
gp19Arf svg 5.1	**CACCG**GGGCCGCCCACTCCAAGAGA	Amplify Cdkn2a locus for Surveyor
gp19Arf svg 3.1	**AAAC**TCTCTTGGAGTGGGCGGCCC**C**	
gKansl2 svg 5.1	CGGATACTGGGTAAGGATCC	Amplify Kansl2 locus for Surveyor
gKansl2 svg 3.1	TAAGGGCAAGACTCAGTTCC	
gIzumo2 svg 5.1	GTGAGGTCACAAAGTCCCCA	Amplify Izumo2 locus for Surveyor
gIzumo2 svg 3.1	TTCTCCCTCTCAACCCTTTC	
gSlmap svg 5.1	AGGCAGTAGACTAGGTGTGA	Amplify Slmap locus for Surveyor
gSlmap svg 3.1	CCTACATTAACACCACAGGC	
gFbxw7 svg 5.1	CCCTGAGAGTAGCAAGCGAA	Amplify Fbxw7 locus for Surveyor
gFbxw7 svg 3.1	TCTTCTGTACTCCCACCCTG	
gFilip1 svg 5.1	ACTGTGATGCTGGTGGACGA	Amplify Filip1 locus for Surveyor
gFilip1 svg 3.1	TTGCCTTATCCTGCTCGGTT	
gFbxo42 svg 5.1	GGCCATTGTGGCACTGTTGT	Amplify Fbxo42 locus for Surveyor
gFbxo42 svg 3.1	CCAGACACAGACTGGGTTCT	
Man2b1 svg 5.1	ACCTATTGGATTCCTGTGGG	Amplify Man2b1 locus for Surveyor
Man2b1 svg 3.1	ACCAGTGCTTCCAGCTGGTT	
gCdkn2b svg 5.1	TAAGCCATTTCTCCGCCCTT	Amplify Cdkn2b locus for Surveyor
gCdkn2b svg 3.1	AACCCGCAGTCAATCTCCAG	
gMat2b svg 5.1	CCCAGTTCTTTAGACAGAGG	Amplify Mat2b locus for Surveyor
gMat2b svg 3.1	CTCCAAGGACCCACATGAAT	
gFam21 svg 5.1	CAGGGCTTTAAATCCCTGTA	Amplify Fam21 locus for Surveyor
gFam21 svg 3.1	CAAGAACCAATCTATCCACC	
gRik svg 5.1	GCTCCTTCCCTGCTTCTAAA	

gRik svg 3.1	TCAGGCACATAGTCAGCTTC	Amplify 4930452B06Rik locus for Surveyor
gObsl1 svg 5.1	TGTATTGGAGAGGCAGGGAG	Amplify Obsl1 locus for Surveyor
gObsl1 svg 3.1	CACCGGAAGCTCTGCACAAA	
gSema4d svg 5.1	CCTGAGTCCTCCTTACACTT	Amplify Sema4d locus for Surveyor
gSema4d svg 3.1	GCGTGACACTTGACTTCTGT	
gCcdc18 svg 5.1	GCTGTGAATATACCTGTGCC	Amplify Ccdc18 locus for Surveyor
gCcdc18 svg 3.1	TGAGCAATACAGTTCAGTGG	
gNfkbil1 svg 5.1	GAGCGTTCAAAACTCCACAC	Amplify Nfkbil1 locus for Surveyor
gNfkbil1 svg 3.1	AGGTTGTCGGAGAGAGGCTA	
gZbtb14 svg 5.1	AGCTAGAAGTAGACAGCTCG	Amplify Zbtb14 locus for Surveyor
gZbtb14 svg 3.1	ACTCCACTTCCTGGCCGTAA	
gArtn svg 5.1	GTGAGTCGCTGGCTTTCCTT	Amplify Artn locus for Surveyor
gArtn svg 3.1	TCACTTTCGGAGGTGTGGGT	
gLrrc8c svg 5.1	CCACTGGTATGCCAAGTACT	Amplify Lrrc8c locus for Surveyor
gLrrc8c svg 3.1	ACTTTGGAAACCAGGGCACT	
gAtm svg 5.1	TCCCATGTGGAAGAAGGGCA	Amplify Atm locus for Surveyor
gAtm svg 3.1	CAAGCATCCAGCAGAGAGAA	
gSlc9a3 svg 5.1	CTAGTCTTGGAGCATGGAAG	Amplify Slc9a3 locus for Surveyor
gSlc9a3 svg 3.1	GGGAGCCTAGCTACTACTAA	

Supplementary Table S4

Top 10 genes co-mutated with *Kras* each type of cancer.

	Gene	Cytoband	Altered group	Unaltered group	Log Ratio	p-Value	q-Value	Tendency
Colon Cancer	*PIK3CA*	3q26.32	752 (24.55%)	531 (13.60%)	0.85	1.45E-31	4.87E-29	Co-occurrence
	APC	5q22.2	2119 (69.18%)	2230 (57.14%)	0.28	2.79E-25	7.02E-23	Co-occurrence
	SMAD4	18q21.2	508 (16.59%)	448 (11.48%)	0.53	5.63E-10	9.43E-08	Co-occurrence
	SMAD2	18q21.1	110 (3.59%)	59 (1.51%)	1.25	1.80E-08	2.58E-06	Co-occurrence
	FBXW7	4q31.3	394 (12.86%)	342 (8.76%)	0.55	2.31E-08	2.90E-06	Co-occurrence
	AMER1	Xq11.2	127 (4.15%)	77 (1.97%)	1.07	7.58E-08	8.46E-06	Co-occurrence
	CTNNB1	3p22.1	171 (5.58%)	166 (4.25%)	0.39	6.16E-03	0.238	Co-occurrence
	TGFBR1	9q22.33	29 (0.95%)	17 (0.44%)	1.12	6.98E-03	0.246	Co-occurrence
	SOX9	17q24.3	243 (7.93%)	253 (6.48%)	0.29	0.0111	0.3	Co-occurrence
	TCF7L2	10q25.2-q	198 (6.46%)	201 (5.15%)	0.33	0.0111	0.3	Co-occurrence
Non Small Cell Lung Cancer	**Gene**	**Cytoband**	**Altered group**	**Unaltered group**	**Log Ratio**	**p-Value**	**q-Value**	**Tendency**
	STK11	19p13.3	605 (19.25%)	595 (7.59%)	1.34	2.68E-64	1.78E-61	Co-occurrence
	ATM	11q22.3	359 (11.42%)	394 (5.03%)	1.18	1.42E-30	4.71E-28	Co-occurrence
	RBM10	Xp11.3	236 (7.51%)	290 (3.70%)	1.02	3.52E-16	9.35E-14	Co-occurrence
	MAX	14q23.3	49 (1.56%)	24 (0.31%)	2.35	9.04E-12	1.33E-09	Co-occurrence
	KEAP1	19p13.2	371 (11.80%)	603 (7.70%)	0.62	1.53E-11	2.04E-09	Co-occurrence
	NKX2-1	14q13.3	81 (2.58%)	73 (0.93%)	1.47	2.75E-10	3.32E-08	Co-occurrence
	U2AF1	21q22.3	72 (2.29%)	70 (0.89%)	1.36	2.05E-08	1.91E-06	Co-occurrence
	IDH1	2q34	63 (2.00%)	63 (0.80%)	1.32	2.97E-07	2.32E-05	Co-occurrence
	AMER1	Xq11.2	76 (2.42%)	87 (1.11%)	1.12	7.02E-07	4.66E-05	Co-occurrence
	NTRK3	15q25.3	167 (5.31%)	268 (3.42%)	0.64	4.77E-06	3.02E-04	Co-occurrence
Pancreatic Cancer	**Gene**	**Cytoband**	**Altered group**	**Unaltered group**	**Log Ratio**	**p-Value**	**q-Value**	**Tendency**
	TP53	17p13.1	1398 (73.93%)	127 (18.76%)	1.98	4.17E-142	3.77E-139	Co-occurrence
	CDKN2A	9p21.3	415 (21.95%)	25 (3.69%)	2.57	6.81E-34	2.05E-31	Co-occurrence
	SMAD4	18q21.2	415 (21.95%)	56 (8.27%)	1.41	3.58E-17	5.40E-15	Co-occurrence
	KDM6A	Xp11.3	80 (4.23%)	9 (1.33%)	1.67	1.04E-04	7.24E-03	Co-occurrence
	RNF43	17q22	99 (5.24%)	14 (2.07%)	1.34	1.83E-04	0.0118	Co-occurrence
	RBM10	Xp11.3	35 (1.85%)	2 (0.30%)	2.65	1.13E-03	0.0513	Co-occurrence
	TGFBR2	3p24.1	48 (2.54%)	5 (0.74%)	1.78	1.91E-03	0.0695	Co-occurrence
	U2AF1	21q22.3	24 (1.27%)	1 (0.15%)	3.1	4.59E-03	0.143	Co-occurrence
	AKT3	1q43-q44	16 (0.85%)	0 (0.00%)	>10	7.35E-03	0.195	Co-occurrence
	SMAD3	15q22.33	32 (1.69%)	3 (0.44%)	1.93	8.37E-03	0.21	Co-occurrence
Soft Tissue Sarcoma	**Gene**	**Cytoband**	**Altered group**	**Unaltered group**	**Log Ratio**	**p-Value**	**q-Value**	**Tendency**
	PIK3CA	3q26.32	7 (21.21%)	69 (3.29%)	2.69	1.12E-04	0.111	Co-occurrence
	KDM5A	12p13.33	4 (12.12%)	19 (0.91%)	3.74	3.43E-04	0.17	Co-occurrence
	SMO	7q32.1	3 (9.09%)	13 (0.62%)	3.88	1.65E-03	0.391	Co-occurrence
	POLE	12q24.33	4 (12.12%)	32 (1.52%)	2.99	1.98E-03	0.391	Co-occurrence
	PARP1	1q42.12	2 (6.06%)	3 (0.14%)	5.41	2.26E-03	0.391	Co-occurrence
	CSF1R	5q32	3 (9.09%)	16 (0.76%)	3.58	2.77E-03	0.391	Co-occurrence
	NTRK2	9q21.33	3 (9.09%)	16 (0.76%)	3.58	2.77E-03	0.391	Co-occurrence
	CHEK1	11q24.2	2 (6.06%)	4 (0.19%)	4.99	3.35E-03	0.415	Co-occurrence
	NOTCH2	1p12	4 (12.12%)	42 (2.00%)	2.6	4.91E-03	0.54	Co-occurrence
	PIK3CD	1p36.22	2 (6.06%)	6 (0.29%)	4.41	6.14E-03	0.608	Co-occurrence
All four cancer types combined	**Gene**	**Cytoband**	**Altered group**	**Unaltered group**	**Log Ratio**	**p-Value**	**q-Value**	**Tendency**
	SMAD4	18q21.2	1018 (12.52%)	771 (5.31%)	1.24	2.42E-79	1.52E-76	Co-occurrence
	APC	5q22.2	2313 (28.45%)	2687 (18.51%)	0.62	1.01E-65	4.76E-63	Co-occurrence
	STK11	19p13.3	679 (8.35%)	662 (4.56%)	0.87	4.27E-30	1.34E-27	Co-occurrence
	CDKN2A	9p21.3	655 (8.06%)	638 (4.40%)	0.87	4.17E-29	1.12E-26	Co-occurrence
	PIK3CA	3q26.32	992 (12.20%)	1152 (7.94%)	0.62	2.35E-25	5.53E-23	Co-occurrence
	ATM	11q22.3	671 (8.25%)	758 (5.22%)	0.66	5.36E-19	1.01E-16	Co-occurrence
	AMER1	Xq11.2	216 (2.66%)	179 (1.23%)	1.11	1.32E-14	2.07E-12	Co-occurrence
	FBXW7	4q31.3	467 (5.74%)	527 (3.63%)	0.66	1.52E-13	2.04E-11	Co-occurrence
	SMAD3	15q22.33	103 (1.27%)	76 (0.52%)	1.27	2.72E-09	2.70E-07	Co-occurrence
	TGFBR1	9q22.33	83 (1.02%)	57 (0.39%)	1.38	1.41E-08	1.26E-06	Co-occurrence
